# Predictive Modeling Using a Somatic Mutational Profile in Ovarian High Grade Serous Carcinoma

**DOI:** 10.1371/journal.pone.0054089

**Published:** 2013-01-10

**Authors:** Insuk Sohn, Chang Ohk Sung

**Affiliations:** 1 Samsung Cancer Research Institute, Seoul, Korea; 2 Department of Pathology, Asan Medical Center, University of Ulsan College of Medicine, Seoul, Korea; Sudbury Regional Hospital, Canada

## Abstract

**Purpose:**

Recent high-throughput sequencing technology has identified numerous somatic mutations across the whole exome in a variety of cancers. In this study, we generate a predictive model employing the whole exome somatic mutational profile of ovarian high-grade serous carcinomas (Ov-HGSCs) obtained from The Cancer Genome Atlas data portal.

**Methods:**

A total of 311 patients were included for modeling overall survival (OS) and 259 patients were included for modeling progression free survival (PFS) in an analysis of 509 genes. The model was validated with complete leave-one-out cross-validation involving re-selecting genes for each iteration of the cross-validation procedure. Cross-validated Kaplan-Meier curves were generated. Cross-validated time dependent receiver operating characteristic (ROC) curves were computed and the area under the curve (AUC) values were calculated from the ROC curves to estimate the predictive accuracy of the survival risk models.

**Results:**

There was a significant difference in OS between the high-risk group (median, 28.1 months) and the low-risk group (median, 61.5 months) (permutated *p*-value <0.001). For PFS, there was also a significant difference in PFS between the high-risk group (10.9 months) and the low-risk group (22.3 months) (permutated *p*-value <0.001). Cross-validated AUC values were 0.807 for the OS and 0.747 for the PFS based on a defined landmark time *t* = 36 months. In comparisons between a predictive model containing only gene variables and a combined model containing both gene variables and clinical covariates, the predictive model containing gene variables without clinical covariates were effective and high AUC values for both OS and PFS were observed.

**Conclusions:**

We designed a predictive model using a somatic mutation profile obtained from high-throughput genomic sequencing data in Ov-HGSC samples that may represent a new strategy for applying high-throughput sequencing data to clinical practice.

## Introduction

Recent high-throughput sequencing technology has generated an enormous amount of data that continues to accumulate for somatic mutations in a variety of cancers. A major issue for these studies is how to separate the useful information from these large volumes of data and how to use the data more effectively. It is important to consider how the data from somatic mutational profiles containing survival information can be applied in clinical use. From this view point, the development of predictive modeling using somatic mutation profiles that employ complete genomic data with survival information may be worthwhile. Predictive modeling has been well studied in microarray gene expression profiling and in proteomic profiling [1], [2], [3]. For predictive modeling, in case where the number of candidate variables exceeds the number of cases, which is common in high throughput genomic data analysis, complete cross-validation is one of established methods and it has widely used for modeling and estimating prediction error in the model [2], [4]. The predictive models are developed from scratch, repeating variable selection and calibration, for each loop of the cross-validation [2]. There are several cross-validation methods, which include leave-one-out cross-validation (LOOCV), v-fold, and bootstrap resampling.

The predictive model with gene signature for predicting patient survival can be used in clinical test. However, no study has attempted to generate a predictive model using a somatic mutational profile obtained from high-throughput sequencing data. Somatic mutation has the potential to reflect patient survival and cancer prognosis. In ovarian high grade serous carcinoma (Ov-HGSC), *BRCA2* mutation is associated with favorable survival and platinum sensitivity [5], [6], [7], [8], [9], [10], [11], [12]. Our previous study revealed that hypermutation in Ov-HGSC patients was associated with platinum sensitivity and favorable survival in patients treated with platinum based chemotherapy after surgery [13]. These findings may suggest that the somatic mutational profile harbors clinical significance and that the combination of specific genes may be predictive of the patient’s survival. Therefore, in this study we generate a predictive model employing the whole exome somatic mutational profile of Ov-HGSCs obtained from The Cancer Genome Atlas (TCGA) data portal.

## Materials and Methods

### Cancer Data

We downloaded a validated whole exome somatic mutation data set and clinical information for 316 patients with Ov-HGSC from TCGA website (http://tcga-data.nci.nih.gov/docs/publications/ov_2001) with open access [12]. Data were downloaded on October 29, 2011. Among the 316 patients, there were a total of 19356 mutations in 9968 genes. Among the 9968 genes, the genes that were very rarely mutated (less than 5 frequencies) were excluded from the selection of genes for modeling. In addition, patients with missing clinical information for overall survival (OS) or progression-free survival (PFS) were excluded from this study. Finally, 311 patients with 509 genes for OS and 259 patients with 509 genes for PFS were included to generate a predictive model for Ov-HGSC. All patients were diagnosed with high-grade serous carcinoma and were in an advanced stage (FIGO stage ≥2). Clinical information including OS, PFS, platinum response status (sensitive vs. resistant), surgical outcome (microscopic residual vs. macroscopic residual), age, and stage were selected. The definition of OS and PFS were described in a previous report [12] and detail clinical informations for each patient were described in a previous report [13].

### Prognostic Model Building and Validation

We used multivariate Cox regression to fit the prediction model. To evaluate the predictive performance, we carried out the LOOCV procedure as follows:

#### Step 1

For the i-*th* sample (i = 1,…,n), divide the i-*th* sample from whole data as the training set and the remaining (n−1) patients as the validation set.

#### Step 2

For the training set, (1) select the genes with log-rank *p*-values <0.01, and standardize the selected gene using the mean and standard deviation of the gene, then (2) apply multivariate Cox regression to the standardized genes.

#### Step 3

For the validation set, (1) standardize each gene using the mean and the standard deviation calculated from the training set, then (2) calculate the risk score as the linear combination of standardized values for each gene and the corresponding coefficient fitted from the multivariate Cox regression.

#### Step 4

Repeat above steps 1–3 for all n samples.

#### Step 5

Dichotomized the predictive scores into low- or high-risk based on median value and cross-validated Kaplan-Meier curves were generated.

To test the statistical significance of the spread between the cross-validated Kaplan-Meier curves, we calculated a permutation *p*-value as follows; (1) compute the log-rank *p*-value (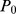) from above LOOCV procedure from the original data, (2) from the b-th permutation data (b = 1,…,B), compute the log-rank *p*-value (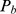) from the LOOCV procedure, and (3) calculate the permutation *p*-value as 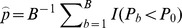.

### Measurement of Predictive Accuracy of Predictive Model

Cross-validated time dependent receiver operating characteristic (ROC) curves using the cross-validated predictive indices were computed to measure the predictive accuracy based on landmark time *t* = 36 months for OS and PFS [2]. ROC curves were generated using the nearest neighbors estimator defined in order to take into account the time of events and the censoring [2], [14], [15]. The area under the curve (AUC) values were calculated from the ROC curves to be used as a measure of predictive accuracy for the survival risk model.

### Statistical Analysis

Cross-validated Kaplan-Meier curves were generated for OS and PFS, and the permutation distribution of the cross-validated log-rank statistic was used for comparing the Kaplan-Meier curves [2]. Chi-square test was used to test the association between the two groups and *p* values less than 0.05 were considered statistically significant. Statistical analysis was performed using Stata/IC statistical software (version 12, StataCorp Ltd., TX) and R program (version 2.12.0: www.r-project.org).

## Results

### Development and Validation of a Prognostic Signature Using Somatic Mutation Data

**Figure 1:**
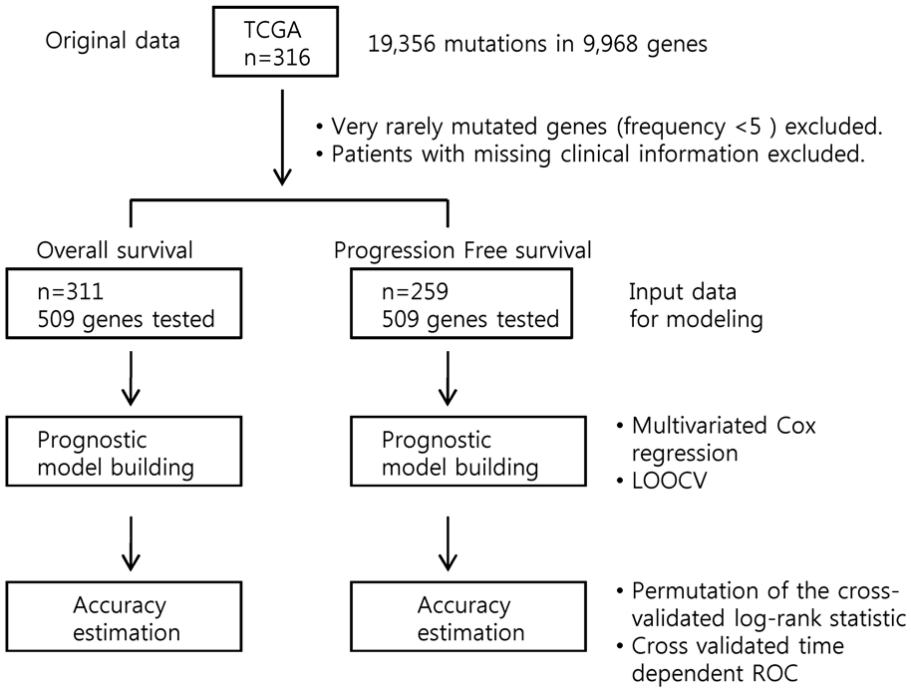
Workflow of prognostic model building using somatic mutation profile in ovarian high-grade serous carcinoma.

**Figure 2:**
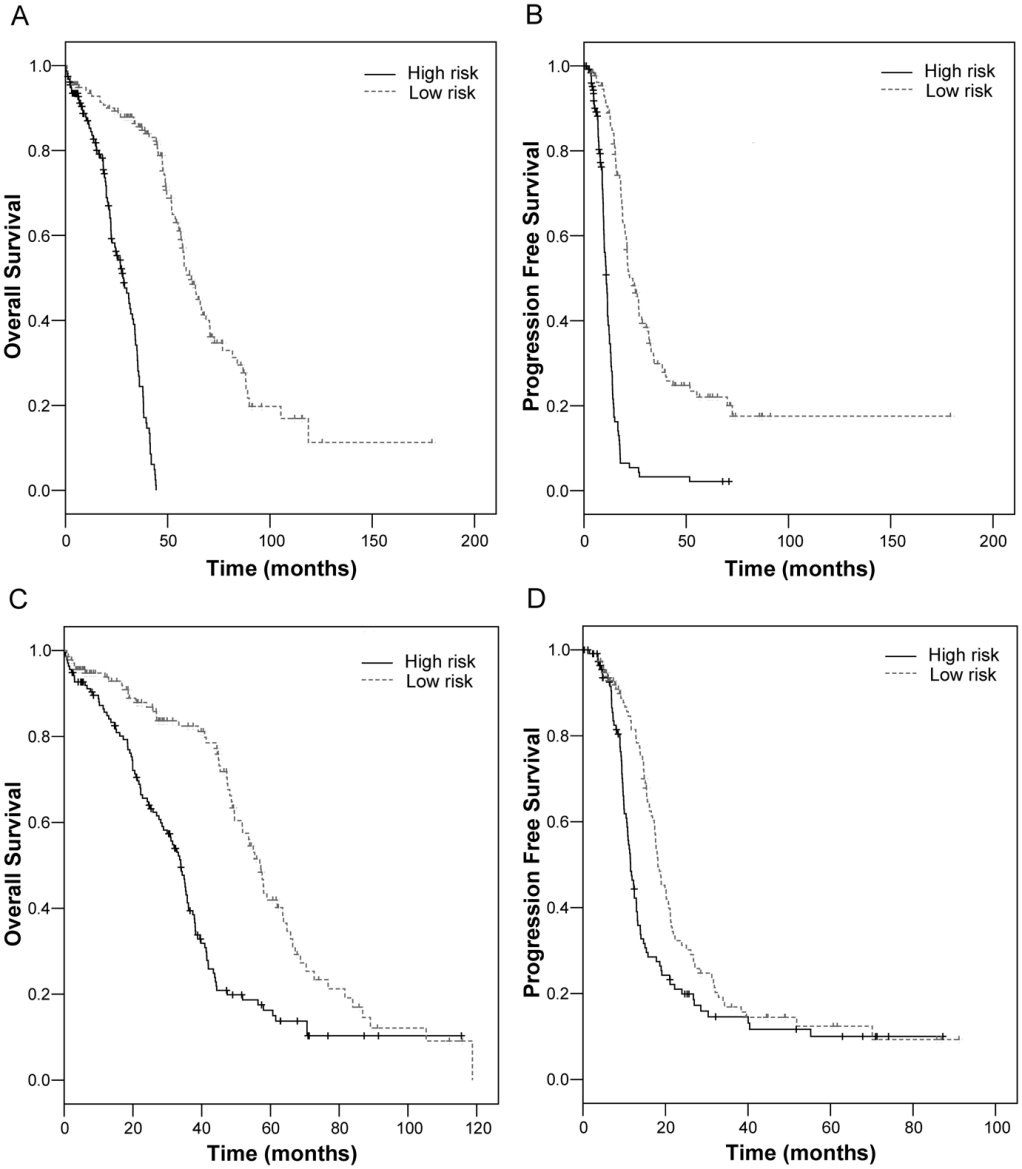
Cross-validated Kaplan-Meier curves of the prognostic models. Model containing only gene variables for overall survival (A) and progression free survival (B). Combined model containing both clinicopathological covariates and gene variables for overall survival (C) and progression free survival (D).

The primary end point was the OS and PFS. The developmental strategy and work flow of predictive models for OS and PFS are schematically depicted in Figure 1. All data presented in this report are based on classification during the LOOCV procedure and are therefore fully cross validated. A permutated *p* value of the cross-validated log-rank statistic was calculated to compare the cross-validated Kaplan-Meier curves. Table 1 summarizes the characteristics of genes selected by fitting Cox proportional hazards models for each OS and PFS. The results of the log-rank test and the frequency of each gene, and the Cox regression estimates used to calculate the score for each gene are shown. Figure 2A shows the cross validated Kaplan-Meier plot for OS based on classification of each case from LOOCV. There was a significant difference in the OS between the high-risk group and the low-risk group (permutated *p*-value <0.001). The median survival time for OS was significantly longer in the low-risk group (61.5 months, 95% CI 55.3–67.7) than in the high-risk group (28.1 months, 95% CI 22.9–33.2) from LOOCV, which corresponds to a hazard ratio for death of 0.077 (95% CI, 0.047–0.125). For PFS, the cross validated Kaplan-Meier plot for PFS is shown in Figure 2B. There was also a significant difference in PFS between the high-risk group and the low-risk group (permutated *p*-value <0.001). The median survival time for PFS was significantly longer in the low-risk group (22.3 months, 95% CI 17.5–27.0) than in the high-risk group (10.9 months, 95% CI 9.9–11.8) from LOOCV, which corresponds to a hazard ratio for recurrence of 0.216 (95% CI, 0.158–0.296).

**Table 1:** Characteristics of genes selected by fitting Cox proportional hazards models.

	Overall survival				Progression free Survival		
Gene	*p*-value	Frequency	Coefficient	Gene	*p*-value	Frequency	Coefficient
*NIPBL*	0.0002	310	0.201804	*BOD1L*	0.0008	259	0.199613
*KIAA0913*	0.0003	309	0.199313	*EPDR1*	0.0000	259	0.042329
*MYH15*	0.0007	309	0.054725	*MYH15*	0.0037	258	0.223415
*NR4A2*	0.0083	309	0.202806	*ACCSL*	0.0016	257	0.096368
*ZNF804B*	0.0020	309	0.135109	*ATM*	0.0026	257	0.284168
*ABCA3*	0.0086	308	0.166472	*OVCH1*	0.0023	257	0.319271
*PIK3R4*	0.0033	308	0.204053	*SORCS3*	0.0022	257	0.164987
*DOCK3*	0.0031	308	0.197207	*CPN2*	0.0050	256	0.19209
				*SPTB*	0.0036	256	0.191474
				*BRCA2*	0.0094	254	−0.20805

### Predictive Accuracy of Models

**Figure 3:**
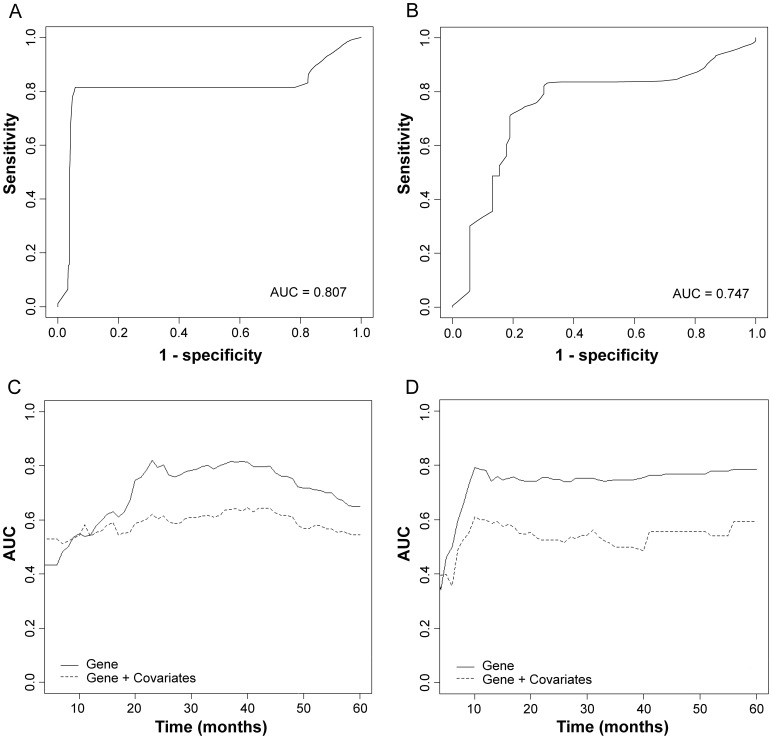
Cross-validated time dependent receiver operating characteristic (ROC) curves and area under the curve (AUC). ROC curve based on landmark time *t* = 36 months of the predictive model for overall survival (A) and progression free survival (B). Cross-validated time dependent AUC curves to compare the prognostic model containing only gene variables with the combined model containing both clinicopathological covariates and gene variables for overall survival (C) and progression free survival (D).

We estimated the predictive accuracy of the model for OS and PFS using time-dependent RO curve. Cross-validated time-dependent ROC curves show the predictive accuracy of the survival risk group model based on a defined landmark time 36 months for OS and PFS in Figure 3A and Figure 3B, respectively. AUC values were 0.807 for the OS and 0.747 for the PFS.

### Association of Risk Groups with Clinicopathological Covariates

Association of risk groups for clinicopathological factors including platinum response status (sensitive vs. resistant), surgical outcome (microscopic vs. macroscopic residual), histological grade (grade 2 vs. 3), tumor stage, and age, are described in Table 2. Among the clinicopathological factors, surgical outcome and platinum response status were significantly associated with risk classification for both OS (*p* = 0.026 and *p* = 0.033, respectively) and PFS (*p*<0.001 and *p*<0.001, respectively).

**Table 2:** Association of risk groups with clinicopathological factors for overall and progression free survival in ovarian high grade serous cancer.

	Overall survival			Progression free survival		
	Lowrisk	Highrisk	*p* value	Lowrisk	Highrisk	*p* value
Age (yrs)						
≤60	78 (50.6)	76 (49.4)	0.956	66 (49.3)	68 (50.7)	0.853
>60	75 (50.3)	74 (49.7)		60 (50.4)	59 (49.6)	
Surgical outcome						
Microscopic	36 (62.1)	22 (37.9)	0.026	31 (62.0)	19 (38.0)	0.033
Macroscopic	99 (45.6)	118 (54.4)		80 (44.9)	98 (55.1)	
Platiunm status,						
sensitive	89 (70.6)	37 (29.4)	<0.001	92 (72.4)	35 (27.6)	<0.001
resistant	18 (29.0)	44 (71.0)		12 (19.4)	50 (80.6)	
Grade						
2	17 (63.0)	10 (37.0)	0.169	14 (66.7)	7 (33.3)	0.119
3	136 (49.1)	141 (50.9)		113 (48.9)	118 (51.1)	
Stage						
2	10 (71.4)	4 (28.6)	0.198	9 (69.2)	4 (30.8)	0.281
3	122 (50.2)	121 (49.8)		104 (50.7)	101 (49.3)	
4	24 (44.4)	30 (55.6)		18 (43.9)	23 (56.1)	

### Comparison to Model Containing Clinicopathological Covariates

To compare predictive model containing only gene variables with combined model containing both clinicopathological covariates and gene variables, we developed a combined survival risk model containing clinicopathological covariates (age ≤60 yrs vs. >60 yrs; grade 2 vs. 3; stages II–III vs. IV; microscopic vs. macroscopic residual surgical outcome) and gene variables. Platinum response status was not included because it cannot be measured at initial surgical treatment. Cross-validated Kaplan-Meier curves of OS (Figure 2C) and PFS (Figure 2D) for the combined survival risk model were generated and the figures show that clinicopathological covariates do not provide additional survival risk discrimination compared to the curves provided by only gene variables. In comparisons with the cross-validated time dependent AUC curves between the predictive model containing only gene variables and the combined model containing both clinicopathological covariates and gene variables (Figure 3C for OS and Figure 3D for PFS), accuracy of the predictive model containing only gene variables is higher than that of the combined model.

## Discussion

The findings in this study demonstrate that predictive modeling using somatic mutational profile obtained from whole exome sequencing has potentially useful applications. Predictive modeling using expression microarray is well established, whereas predictive modeling using mutational profiling has not been attempted until now. Most of the differences between microarray data and somatic mutation data are characteristics of value. Expression microarray data has continuous value, whereas a somatic mutation profile contains binary data. In other words, expression microarray data is a relative value, whereas somatic mutation data is an absolute value that indicates the presence or absence of mutation. Therefore, if modeling using somatic mutation data is well established, it may be more predictive for independent external samples.

Currently, high-throughput sequencing in cancer is being employed more often, and large amount of data from sequencing will be generated. Therefore, our approach for generating predictive modeling using somatic mutational profiles obtained from high-throughput sequencing could be an effective method in the high-throughput sequencing era.

A few noteworthy issues arose while creating the predictive model from the somatic mutation data in this study. We acknowledge that the genes considered in the modeling may include passenger mutations. Currently, a major question is that what is a driver mutated gene in the numerous mutated genes. This was also a major issue that needed to be addressed before making the predictive model using somatic mutation profiles. Together, these issues may result in non-reproducible results in external independent data set. In our present analysis, one factor underlying the construction of a successful predictive model may have been the large sample size, despite the rare frequency of repeated mutated genes. In the future, it may be necessary to develop additional algorithms that are adjusted to fit somatic mutational profiles in cases of relatively small sample sizes. On the other hand, the integration of a mutation profile, copy number change, and gene expression profile with the goal of generating a predictive model may be a more promising approach.

There are several methods for predictive modeling and estimation of the predictive model [1], [4], [16], [17], [18]. In this study, we used complete LOOCV for predictive model, and permutation distribution of the cross-validated log-rank statistic was used for comparing the cross-validated Kaplan-Meier curves and evaluated the accuracy of the predictive model according to the study by Simon et al [2]. This complete cross-validation method avoids optimistic bias in estimation of survival risk discrimination for the survival risk model developed on the full data set [2].

Among the genes selected by fitting Cox proportional hazards models for PFS, the *BRCA2* mutation was associated with favorable survival, which was well demonstrated form the original TCGA study as well as several other studies [8], [13], [19], [20]. All of the remaining genes, excluding *BRCA2,* were associated with poor prognosis, which was not identified in previous studies. Even though this study included some already well-known genes such as *ATM* [21], most of the genes have not been extensively studied and may need further validation.

In Ov-HGSC, the standard treatment is aggressive tumor cytoreductive surgery followed by platinum based chemotherapy. Ov-HGSC is usually platinum-sensitive [12], [22], [23]. However, approximately 30% of patients exhibit platinum resistance and aggressive disease progression [13], [24], [25]. However, it is difficult to predict survival in the patient with Ov-HGSC after initial standard surgical treatment. Our present study showed that predictive models containing only gene mutation profile without clinical covariates were effective and high AUC values for both OS and PFS were observed.

In conclusion, we designed a predictive model using a somatic mutation profile obtained from high-throughput genomic sequencing data for Ov-HGSC samples that may represent a new strategy for applying high-throughput sequencing data to clinical practice.

## References

[1] Simon R, Radmacher MD, Dobbin K, McShane LM (2003) Pitfalls in the use of DNA microarray data for diagnostic and prognostic classification. J Natl Cancer Inst 95: 14–18. 12509396 10.1093/jnci/95.1.14

[2] Simon RM, Subramanian J, Li MC, Menezes S (2011) Using cross-validation to evaluate predictive accuracy of survival risk classifiers based on high-dimensional data. Brief Bioinform 12: 203–214. 21324971 10.1093/bib/bbr001 PMC3105299

[3] Waldron L, Pintilie M, Tsao MS, Shepherd FA, Huttenhower C, et al (2011) Optimized application of penalized regression methods to diverse genomic data. Bioinformatics 27: 3399–3406. 22156367 10.1093/bioinformatics/btr591 PMC3232376

[4] Molinaro AM, Simon R, Pfeiffer RM (2005) Prediction error estimation: a comparison of resampling methods. Bioinformatics 21: 3301–3307. 15905277 10.1093/bioinformatics/bti499

[5] Moynahan ME, Pierce AJ, Jasin M (2001) BRCA2 is required for homology-directed repair of chromosomal breaks. Mol Cell 7: 263–272. 11239455 10.1016/s1097-2765(01)00174-5

[6] Patel KJ, Yu VP, Lee H, Corcoran A, Thistlethwaite FC, et al (1998) Involvement of Brca2 in DNA repair. Mol Cell 1: 347–357. 9660919 10.1016/s1097-2765(00)80035-0

[7] Xia F, Taghian DG, DeFrank JS, Zeng ZC, Willers H, et al (2001) Deficiency of human BRCA2 leads to impaired homologous recombination but maintains normal nonhomologous end joining. Proc Natl Acad Sci U S A 98: 8644–8649. 11447276 10.1073/pnas.151253498 PMC37489

[8] Yang D, Khan S, Sun Y, Hess K, Shmulevich I, et al (2011) Association of BRCA1 and BRCA2 mutations with survival, chemotherapy sensitivity, and gene mutator phenotype in patients with ovarian cancer. JAMA 306: 1557–1565. 21990299 10.1001/jama.2011.1456 PMC4159096

[9] Foulkes WD (2006) BRCA1 and BRCA2: chemosensitivity, treatment outcomes and prognosis. Fam Cancer 5: 135–142. 16736282 10.1007/s10689-005-2832-5

[10] Norquist B, Wurz KA, Pennil CC, Garcia R, Gross J, et al (2011) Secondary somatic mutations restoring BRCA1/2 predict chemotherapy resistance in hereditary ovarian carcinomas. J Clin Oncol 29: 3008–3015. 21709188 10.1200/JCO.2010.34.2980 PMC3157963

[11] Yuan SS, Lee SY, Chen G, Song M, Tomlinson GE, et al (1999) BRCA2 is required for ionizing radiation-induced assembly of Rad51 complex in vivo. Cancer Res 59: 3547–3551. 10446958

[12] The Cancer Genome Atlas Research Network (2011) Integrated genomic analyses of ovarian carcinoma. Nature 474: 609–615. 21720365 10.1038/nature10166 PMC3163504

[13] Sohn I, Jung WY, Sung CO (2012) Somatic hypermutation and outcomes of platinum based chemotherapy in patients with high grade serous ovarian cancer. Gynecol Oncol 126: 103–108. 22484402 10.1016/j.ygyno.2012.03.050

[14] Heagerty PJ, Zheng Y (2005) Survival model predictive accuracy and ROC curves. Biometrics 61: 92–105. 15737082 10.1111/j.0006-341X.2005.030814.x

[15] Heagerty PJ, Lumley T, Pepe MS (2000) Time-dependent ROC curves for censored survival data and a diagnostic marker. Biometrics 56: 337–344. 10877287 10.1111/j.0006-341x.2000.00337.x

[16] Azuaje F (2003) Genomic data sampling and its effect on classification performance assessment. BMC Bioinformatics 4: 5. 12553886 10.1186/1471-2105-4-5 PMC149349

[17] Braga-Neto UM, Dougherty ER (2004) Is cross-validation valid for small-sample microarray classification? Bioinformatics 20: 374–380. 14960464 10.1093/bioinformatics/btg419

[18] Hickey JM, Veerkamp RF, Calus MP, Mulder HA, Thompson R (2009) Estimation of prediction error variances via Monte Carlo sampling methods using different formulations of the prediction error variance. Genet Sel Evol 41: 23. 19284698 10.1186/1297-9686-41-23 PMC3225835

[19] Gallagher DJ, Konner JA, Bell-McGuinn KM, Bhatia J, Sabbatini P, et al (2011) Survival in epithelial ovarian cancer: a multivariate analysis incorporating BRCA mutation status and platinum sensitivity. Ann Oncol 22: 1127–1132. 21084428 10.1093/annonc/mdq577 PMC6267858

[20] Lacour RA, Westin SN, Meyer LA, Wingo SN, Schorge JO, et al (2011) Improved survival in non-Ashkenazi Jewish ovarian cancer patients with BRCA1 and BRCA2 gene mutations. Gynecol Oncol 121: 358–363. 21276604 10.1016/j.ygyno.2010.12.354 PMC3310886

[21] McConville CM, Stankovic T, Byrd PJ, McGuire GM, Yao QY, et al (1996) Mutations associated with variant phenotypes in ataxia-telangiectasia. Am J Hum Genet 59: 320–330. 8755918 PMC1914715

[22] Bowtell DD (2010) The genesis and evolution of high-grade serous ovarian cancer. Nat Rev Cancer 10: 803–808. 20944665 10.1038/nrc2946

[23] Sabatier R, Finetti P, Cervera N, Birnbaum D, Bertucci F (2009) Gene expression profiling and prediction of clinical outcome in ovarian cancer. Crit Rev Oncol Hematol 72: 98–109. 19249225 10.1016/j.critrevonc.2009.01.007

[24] Bookman MA (2003) Developmental chemotherapy and management of recurrent ovarian cancer. J Clin Oncol 21: 149s–167s. 17633784 10.1200/jco.2003.02.553

[25] Miller DS, Blessing JA, Krasner CN, Mannel RS, Hanjani P, et al (2009) Phase II evaluation of pemetrexed in the treatment of recurrent or persistent platinum-resistant ovarian or primary peritoneal carcinoma: a study of the Gynecologic Oncology Group. J Clin Oncol 27: 2686–2691. 19332726 10.1200/JCO.2008.19.2963 PMC2690393

